# Natural variation in the maternal and zygotic mRNA complements of the early embryo in *Drosophila melanogaster*

**DOI:** 10.1186/s12864-022-08839-4

**Published:** 2022-09-08

**Authors:** Anna A. Feitzinger, Anthony Le, Ammon Thompson, Mehnoor Haseeb, Mohan Koumar Murugesan, Austin M. Tang, Susan E. Lott

**Affiliations:** Department of Evolution and Ecology, University of California, Davis, CA 95616 USA

**Keywords:** Early embryonic development, Maternal gene expression, Early zygotic gene expression, Expression variation

## Abstract

**Background:**

Maternal gene products supplied to the egg during oogenesis drive the earliest events of development in all metazoans. After the initial stages of embryogenesis, maternal transcripts are degraded as zygotic transcription is activated; this is known as the maternal to zygotic transition (MZT). Recently, it has been shown that the expression of maternal and zygotic transcripts have evolved in the Drosophila genus over the course of 50 million years. However, the extent of natural variation of maternal and zygotic transcripts within a species has yet to be determined. We asked how the maternal and zygotic pools of mRNA vary within and between populations of *D. melanogaster.* In order to maximize sampling of genetic diversity, African lines of *D. melanogaster* originating from Zambia as well as DGRP lines originating from North America were chosen for transcriptomic analysis.

**Results:**

Generally, we find that maternal transcripts are more highly conserved, and zygotic transcripts evolve at a higher rate. We find that there is more within-population variation in transcript abundance than between populations and that expression variation is highest post- MZT between African lines.

**Conclusions:**

Determining the natural variation of gene expression surrounding the MZT in natural populations of *D. melanogaster* gives insight into the extent of how a tightly regulated process may vary within a species, the extent of developmental constraint at both stages and on both the maternal and zygotic genomes, and reveals expression changes allowing this species to adapt as it spread across the world.

**Supplementary Information:**

The online version contains supplementary material available at 10.1186/s12864-022-08839-4.

## Background

Over the course of the development of multicellular organisms, an embryo that starts with a single nucleus undergoes divisions with dynamic changes in gene expression to give rise to a functional organism. This can require tight temporal and spatial control of gene expression throughout development, which is complicated by the fact that early development requires the coordination of gene expression across two different genomes [1]. The earliest steps of embryonic development are under complete control of gene products supplied by the maternal genome before developmental control is transferred to the zygote [1]⁠⁠⁠. This process, where control of development is handed off between the maternal and zygotic genomes, is known as the maternal to zygotic transition (MZT) and has been the subject of study of many model organisms [2]⁠. In *Drosophila melanogaster*, maternal RNAs are transcribed during oogenesis in specialized cells called nurse cells and then supplied to the oocyte [3]⁠. During the MZT, these maternal RNAs are degraded as the zygotic genome is activated, ~ 2.5 h after fertilization [4]⁠. Levels of many transcripts produced by both the maternal and zygotic genomes appear invariant across the MZT, indicating precise coordination of maternal degradation and zygotic transcription [5]⁠.

Given the importance of early development to organism survival and its dependence on precise regulation and coordination across the maternal and zygotic genomes, it may be unsurprising that a previous study found a high level of conservation of transcript levels at these stages across *Drosophila* species [6]⁠. However, the same study [6]⁠ also identified changes in transcript representation and abundance across the 50 million years of divergence time of *Drosophila* at both the maternal and zygotic stages. Given that these species have significant differences in the environments in which they develop, some of these changes may be functionally critical to developing under different conditions. Correlations of maternal and zygotic transcript levels decreased with evolutionary divergence, and changes in transcript representation were found even between closely related species [6]⁠. Yet, a significant question remains: do differences in maternal and zygotic transcript levels evolve in the comparatively short evolutionary timescales represented by different populations within a species? Understanding the extent of changes in transcript levels in these critical developmental stages of populations within a species can inform us about the timescale of evolutionary change. Exploring the types of genes that change in the context of different populations may also be a promising avenue for understanding the functions and potential adaptive value of these changes.

In this study, we sought to determine the extent of variation in maternal and zygotic embryonic transcriptomes within and between populations. To maximize the probability of observing differences, we chose populations of *D. melanogaster* from Africa and North America, as these were likely to be highly genetically diverged. As a species, there is evidence that *D. melanogaster* has its origins in Sub-Saharan Africa [7, 8]⁠⁠. Approximately 10,000 years ago, it is likely that *D. melanogaster* began to expand beyond Sub-Saharan Africa and eventually into northern Africa, Asia, and Europe [9, 10]⁠. Only within the past few hundred years were North American populations of *D. melanogaster* founded [11]. With the expansion of *D. melanogaster* out of Sub-Saharan Africa, there was likely a significant loss in genetic diversity ⁠[12]⁠. Efforts to sequence genomes from different lines and geographic populations of *D. melanogaster*, including African populations, has been ongoing in order to understand underlying genetic variation and the demographic history of the species [8]⁠.⁠ Taking advantage of the large number of sequenced genomes and RNA sequencing technology, it has more recently become possible to interrogate correlations between genetic variation and transcriptome diversity. For instance, a previous study found that for adult flies, the greater genetic diversity of African populations of *D. melanogaster* did not result in a significantly higher level of gene expression differences within an African population as compared to within a European population ⁠[13]⁠. This has brought to light the extent of differential gene expression between these populations within the same species.

Here, we address how the maternal and zygotic transcriptomes controlling the critical processes in early embryogenesis differ within and between populations of *D. melanogaster*. We performed RNA-Seq on embryos from four lines from Zambia and four lines from North America, from two developmental stages, one stage where all transcripts present are maternal in origin and the other after zygotic genome activation. Transcript level variation was quantified within two populations as well as putative fixed differences in gene expression between them. We discovered that variation of both maternal and zygotic transcript levels is higher within populations than between populations. We find that there is more expression variation within the Zambia population at both stages relative to the Raleigh (North Carolina, USA) population. We observe an enrichment on the X chromosome for maternally deposited mRNAs that are differentially deposited between the two populations. Additionally, we find less transcript level variation between any two of our *D. melanogaster* lines than between species of *Drosophila* ranging 250,000—8 million years divergence time (*D.simulans* versus *D.sechellia* and *D. yakuba* versus *D.erecta*). Overall, our results demonstrate that expression level variation at these two stages is consistent with what is known about the differences in genetic variation between these populations. Furthermore, differences in transcript levels at these two stages between populations of *D. melanogaster* recapitulate what is known between species of *Drosophila.*

## Results

**Fig.1:**
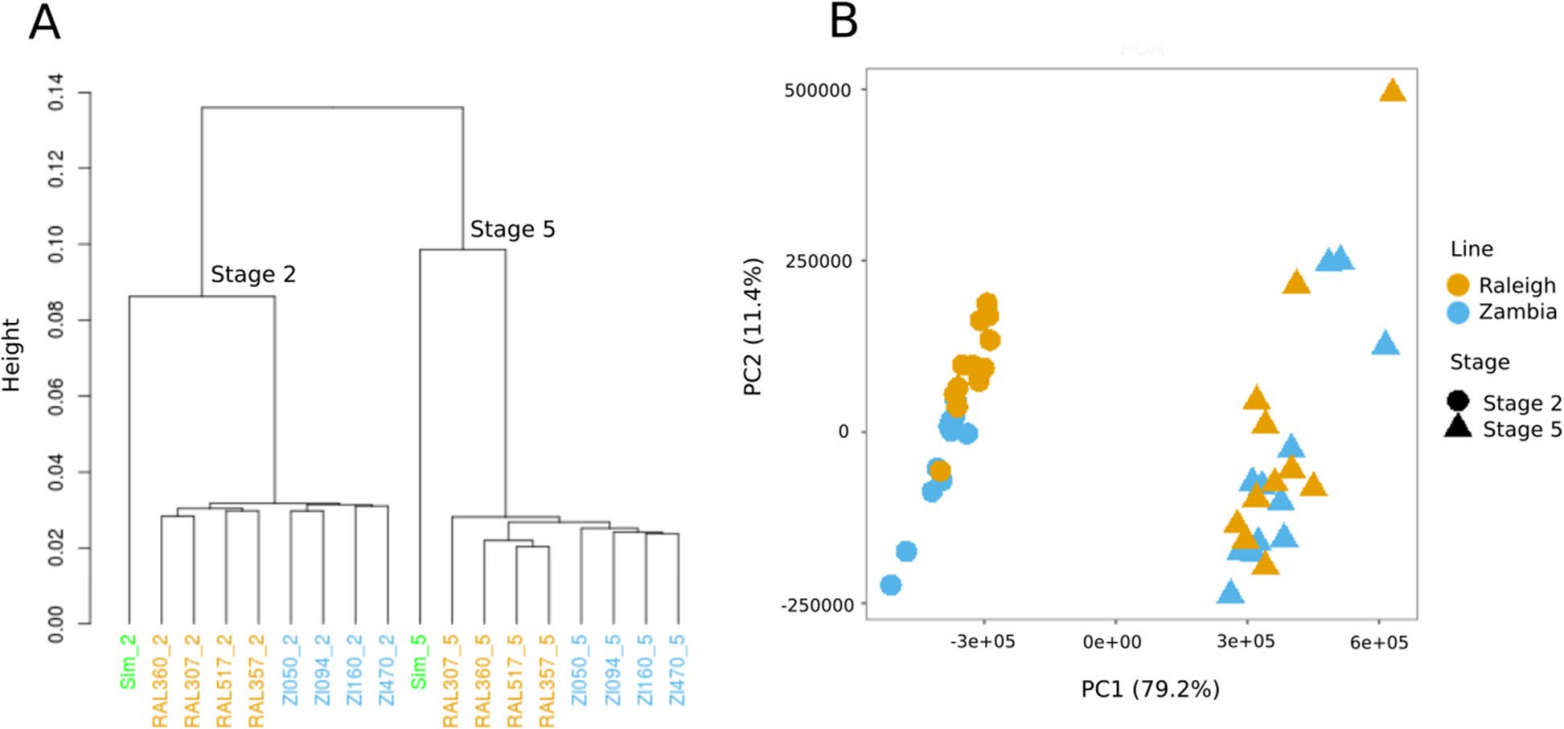
Populations are distinct at each developmental stage. **A** Hierarchical clustering of transcriptomes from stage 2 (labels ending with _2) and stage 5 (labels ending with _5) embryos, from 8 lines of *D. melanogaster*, four from Raleigh (RAL, orange) and four from Zambia (ZI, blue), with closely related species *D. simulans* (Sim, in green text) as an outgroup. Samples cluster first by stage, then by species, then by population. **B** PCA shows that these same samples separate first by stage (PC1, which explains a large proportion of the variance at 79.2%), then by population (PC2, 11.4% of the variance), though more distinctly at stage 2 than stage 5

In order to investigate the natural variation of RNA levels within a species at stages of embryogenesis controlled by maternal and zygotic genomes, we sequenced embryonic transcriptomes from different *D. melanogaster* populations. Single embryos were collected at a stage in which all RNA has been maternally provided (Bownes’ stage 2, [14]⁠⁠), and another stage after zygotic genome activation (late stage 5; or end of blastoderm stage). In order to maximize genetic diversity, we chose four lines from Siavonga, Zambia [15] and four Drosophila Genetics Reference Panel (DGRP) [16]⁠⁠ lines from Raleigh, North Carolina. Three biological replicates were sequenced per line and stage. An average of 2.83 and 2.89 million high-quality 100 bp paired-end reads were mapped to the same reference *D. melanogaster* genome from the Zambia and Raleigh lines, respectively. Hierarchical clustering of the transcriptomes resulted in samples clustering initially by stage then by population, with the exception of one Raleigh line whose stage 5 sample fell outside the three other stage 5 Raleigh samples (Fig. 1A). When we included transcriptomes from an outgroup, *D. simulans,* which share a common ancestor ~ 2.5 MYA with *D. melanogaster* [17]⁠⁠, to the clustering, the *D. simulans* samples clustered by stage with, but outside of, the *D. melanogaster* transcriptomes (Fig. 1A). Principal component analysis also separates individual lines by stage with the corresponding principal component (PC1) representing nearly 80% of the variation (Fig. 1B).

### Expression variation differs within populations

**Fig. 2:**
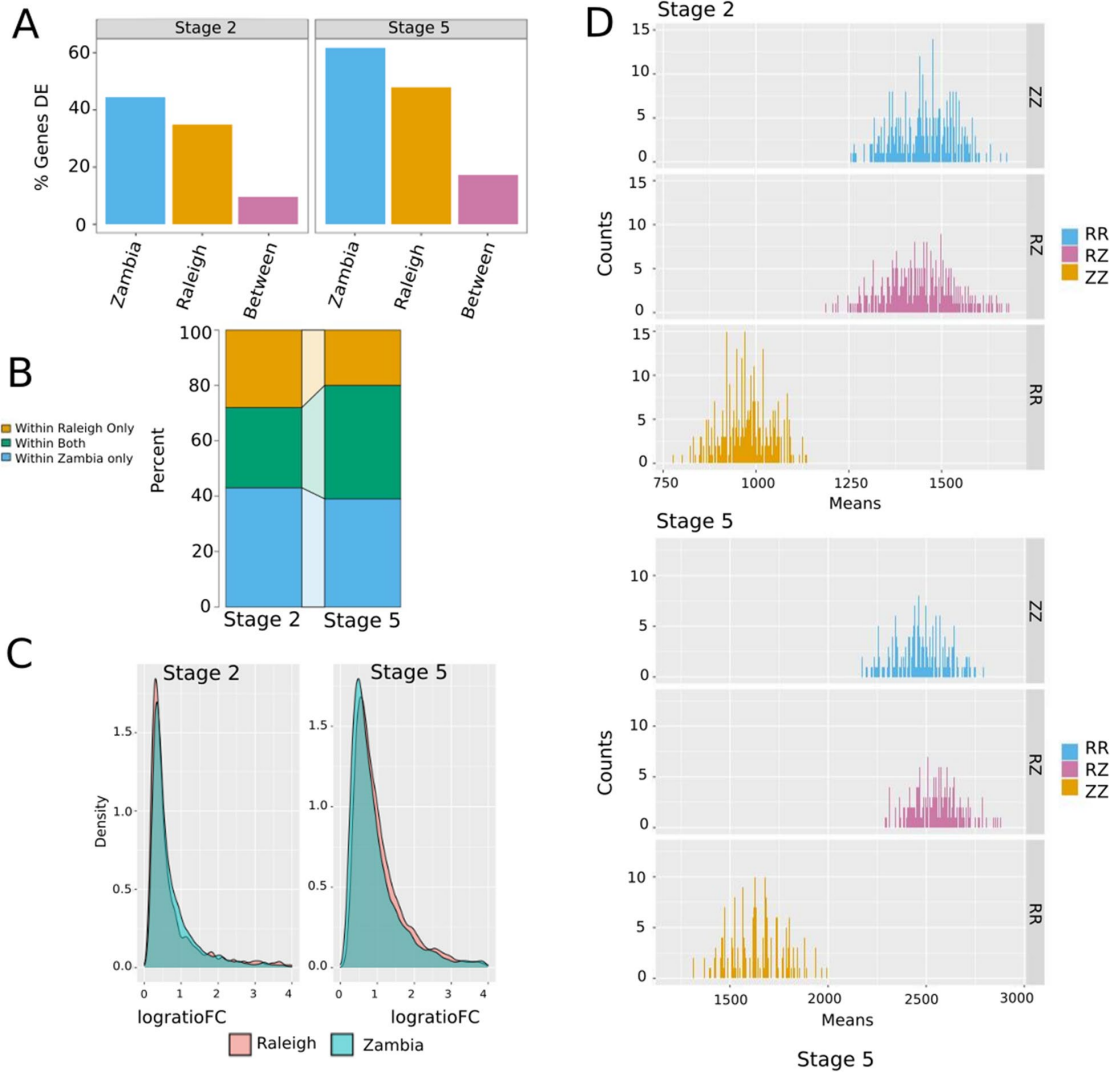
Differential expression within and between populations (Higher number of differential expression within Zambia. Larger magnitude of changes within Raleigh at Stage 5) **A** Percent of genes differentially expressed within and between the Zambia and Raleigh populations at stage 2 and stage 5. More differences are found within populations (blue, orange) than between populations (pink). **B** Proportion of transcripts which have variation in transcript levels in only one population (orange or blue) or have differentially expression within both populations (green) at stage 2 and stage 5. **C** To control for the number of comparisons within and between lines, we also examined pairwise differences between lines at each stage (stage 2 top, stage 5 bottom). When compared in this way, at both stages, the distributions of DE genes within the Zambia population and between the Zambia and Raleigh populations are similar, with fewer DE genes within the Raleigh lines. **D** Distributions of the magnitudes of differences in expression in DE genes, which shows that the magnitude of changes between differentially expressed genes is greater within the Raleigh population at stage 5 than the Zambia population at this stage

To explore the patterns of variation in the maternal and zygotic embryonic transcriptomes within and between populations of *D. melanogaster*, we performed differential expression (DE) analysis on our transcriptomic dataset. First, we asked how many genes are differentially expressed within each population, Zambia and Raleigh, at maternal and zygotic stages of development. To do this, we implemented a likelihood ratio test in DESeq2. We normalized our differential expression results to numbers of genes expressed (see Methods) at each stage in order to compare proportions of genes differentially expressed between stages. We found that overall, there are more differentially expressed genes at stage 5 than at stage 2 within both populations (Fig. 2A). This is consistent with previous findings between species that zygotic gene expression evolves faster than maternal gene expression [6]⁠. Strikingly, there are many more differentially expressed genes at both stage 2 and stage 5 within the Zambia population than between Raleigh lines. Total number of DE genes within these populations were 3174 and 2512 at stage 2, respectively. At stage 5 total number of DE genes was 4723 within the Zambian population and 3648 within the Raleigh population.

We asked if there were similarities in the identity of genes with differential expression within populations at the two stages. A proportion of genes were found to be differentially expressed within both populations at stage 2 and stage 5 (Fig. 2A). Of all the DE genes at stage 2 combined, 43% were only DE within Zambia and 28% within only Raleigh lines, while 29% of genes were DE in both (Fig. 2B). At stage 5 the percent of genes only DE within the Zambia lines stayed relatively similar at 39% whereas the percentage of genes only varying expression within the Raleigh population was lower at 20%, due to a higher proportion of genes in both (at 41%; Fig. 2B). Thus, the percentage of genes varying in expression levels in both populations is higher in stage 5 than stage 2. Therefore, there is a common set of genes that vary in transcript levels within both populations in addition to a unique set of genes that vary only within the respective populations, and these vary by stage, with more shared differences at stage 5.

### Differences in the magnitude of expression variation within populations

With more genes differentially expressed within the Zambia population than the Raleigh population, we asked if the magnitude of expression changes were similar between populations. To do this, we used the maximum and minimum expression value for each differentially expressed gene within the populations. From this, we computed the log ratio of the fold change for each DE gene. We then asked if the distribution of the log ratio of fold changes for DE genes were different between the two populations at either stage (Fig. 2C). There is no significant difference between the means of log ratio of fold changes when comparing stage 2 between populations (t-test, *p* = 0.9109), thus no evidence that the magnitude of transcript abundance changes is different between populations. There is, however, a significant difference between the means of the log ratio of fold changes between the two populations at stage 5 (t-test, *p* = 7.278e-06) with a higher magnitude of fold changes within the Raleigh population. Therefore, although there are fewer genes differentially expressed within the Raleigh population at stage 5, the magnitude of these differences are on average higher than the genes differentially expressed within the Zambia population at this stage.

### More differences within populations than between populations at maternal and zygotic stages

**Fig. 3:**
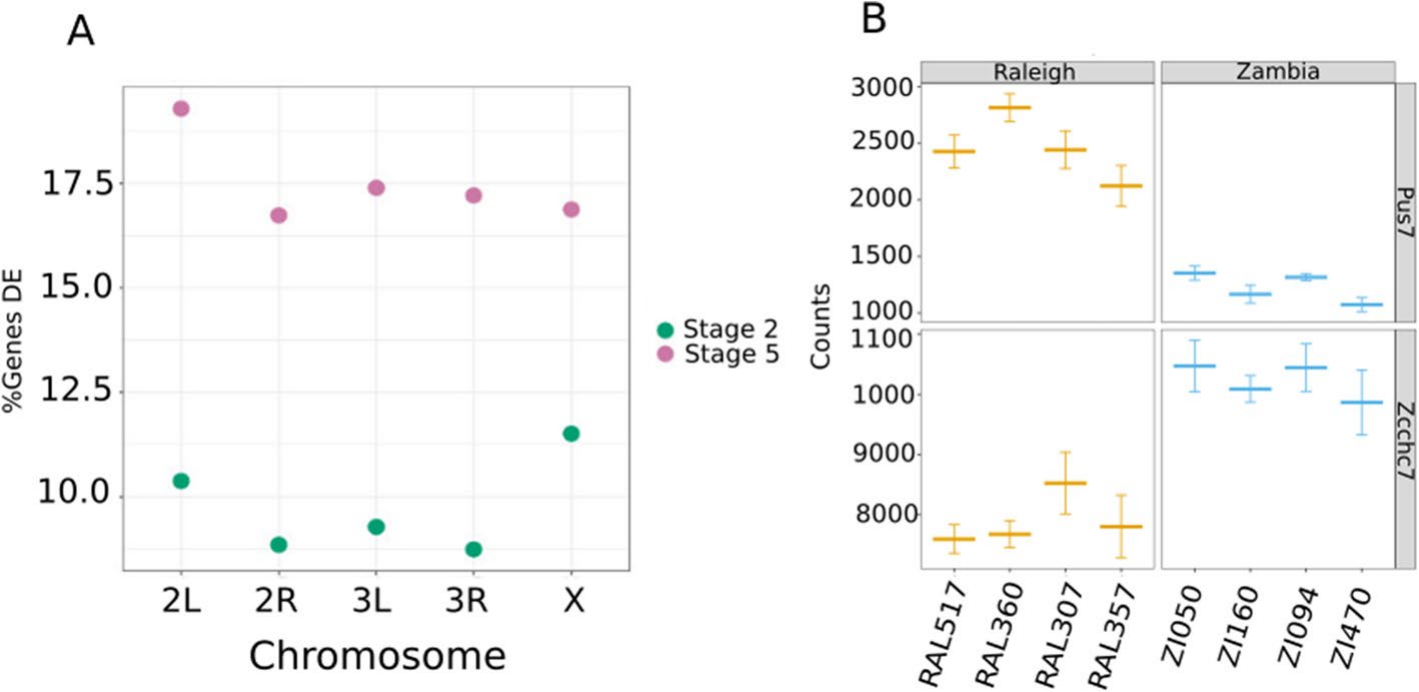
Examination of putative fixed differences between populations. **A** Expression levels in counts for two example genes, showing what we categorize as fixed differences in transcript levels between populations. This is an example of the category of genes summarized in panel B. **B** Percentage of genes that are differentially expressed between populations as compared to the number of genes on the chromosome at each stage. At stage 2, where all transcripts are maternal in origin, there is a significant enrichment of DE genes on the X chromosome

Next, we asked if there were fixed expression differences between the populations. We define fixed expression differences as genes that are on average higher, or lower, in one population than the other (i.e. have similar levels in all lines from a population, that are significantly different than all the lines in the other population; see Fig. 3A for examples). We used the Raleigh lines and the Zambia lines as replicates in DE analysis. Similar to the expression variation within populations, the percentage of genes that were differentially expressed between populations increased from stage 2 to stage 5 (Fig. 2A). We find that there are more genes differentially expressed within populations than fixed expression differences between the populations at both stages (Fig. 2A). We find 700 and 1325 genes differentially expressed between populations at stage 2 and stage 5, respectively.

In addition to finding fixed expression differences, we asked how many genes were differentially expressed between individual lines. Genes differentially expressed between lines from different populations in the pairwise analysis represent differences only between the two lines in the comparison, rather than fixed expression differences between the two populations as in the previous analysis. This resulted in DE analysis between every pair of lines resulting in 28 of total comparisons. 12 DE tests between lines of the same population (RR and ZZ), and 16 DE tests between lines of different populations (RZ) (Fig. 2D). Since there are fewer tests between lines of the same population than between lines of different populations we used bootstrapping in order to compare the average number of DE genes between these categories. Similarly to the previous within population analysis, there are fewer DE genes between individual Raleigh lines (RR) than Zambia lines (ZZ), at both stages (Fig. 2D). We find that the average pairwise differences between lines (RZ) of different populations at stage 2 was not significantly different (*p* = 0.06972; Wilcoxon rank sum test) than the average pairwise differences between Zambia lines (ZZ) at this stage (Fig. 2D). However, at stage 5, the average number of differences between lines of different populations are higher relative to the number of differences between Zambia lines (*p* < 2.2e-16; Wilcoxon rank sum test). Therefore, there is as much variation of expression between individual Zambia lines at stage 2 as between individual lines from different populations at this stage. In contrast, variation between individual lines from different populations at stage 5 surpasses the differences between individual Zambia lines at this stage.

### More expression variation between than within species

**Fig. 4:**
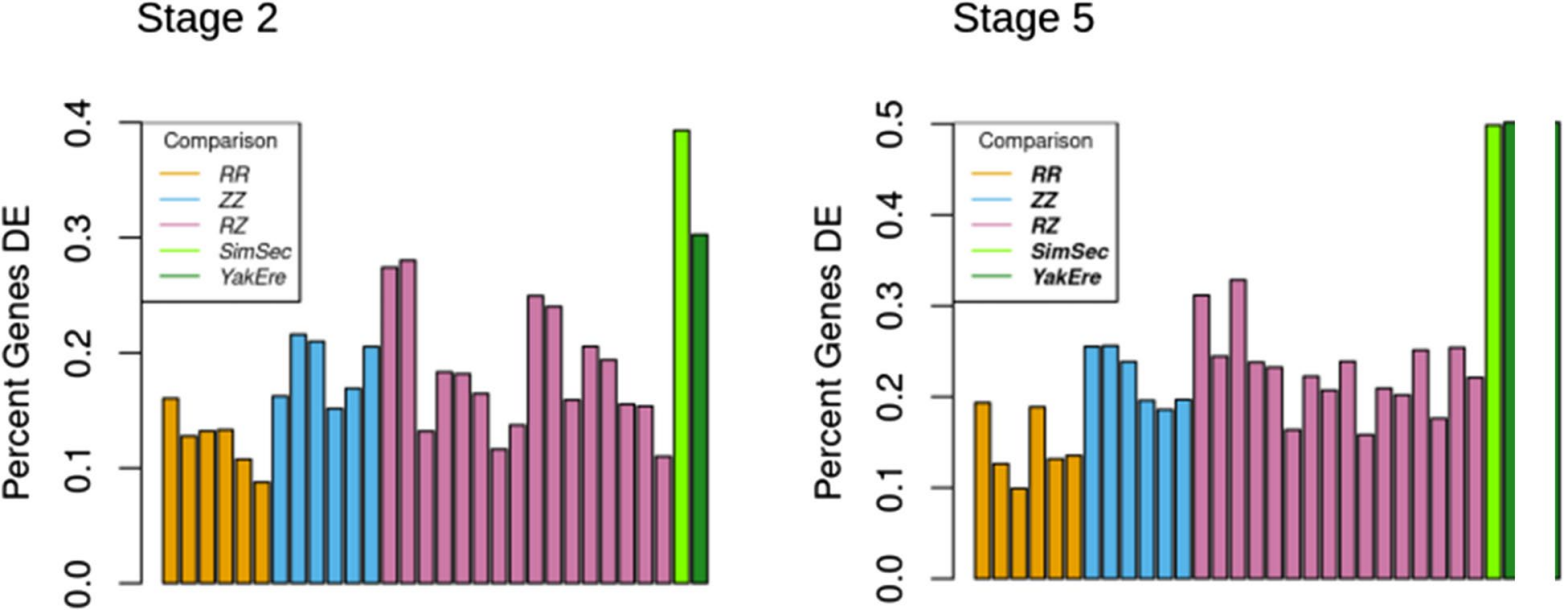
Differential Expression within and Between Species. DE analysis was done between individual Raleigh lines (orange), individual Zambia lines (blue), between lines of the two populations (purple) and between species pairs (green) from stage 2 and stage 5 embryos. The between species DE analysis was done between *D. simulans* and *D. sechellia* as well as *D. yakuba* and *D.erecta.* It was found that there were on average fewer DE genes between lines of *D. melanogaster* than between specie pairs at both stages

Expanding our analysis, we investigated gene expression variation within and between species of *Drosophila* at maternal and zygotic stages. In a previous study, we generated RNA-seq data from *D. simulans, D. sechellia, D. yakuba,* and *D. erecta* from stage 2 and stage 5 embryos using the same single embryo RNA extraction method implemented here. We chose these two pairs of sister species as they are closely related, but one pair (*D. simulans* and *D. sechellia*) diverged more recently, ~ 250,000 years ago, [17]⁠⁠ than the other pair (*D. yakuba* and *D. erecta,* estimated 8 MYA divergence time [18, 19]⁠⁠. RNA-seq reads from these species were processed identically to the *D. melanogaster* reads for this analysis (see Methods). Genes considered in this analysis were limited to one-to-one orthologs across the 5 species, a total of 12,110 genes. As we had only one line for each of the other species, we performed the DE analysis pairwise for each of our *D. melanogaster* lines, as well as between each pair of sister species. The number of DE genes in each population or species was normalized to the number of genes transcribed at each stage to compare the percentage of DE genes at both stages and across species. From these comparisons, within and between species, there are more differentially expressed genes at stage 5 than stage 2 (Fig. 4). For maternal genes, the more closely related species pair between the two species pairs, *D. simulans* and *D. sechellia* have the highest proportion of DE genes. While most *D. melanogaster* lines have fewer differences than either of the species comparisons at this stage, two of the Raleigh vs. Zambia comparisons have as high of a proportion of their maternal genome differentially expressed as the more distantly related species pair, *D. yakuba* and *D. erecta*. For stage 5, both species pairs have a larger proportion of their transcripts DE than any of the within-species comparisons of *D. melanogaster.* Both stages have, on average, fewer genes DE for within-species comparisons than between species, but this pattern is much stronger for stage 5, a stage with more genes DE in all comparisons.

### Enrichment of DE genes at maternal stage on the X chromosome

Before the zygotic genome is activated, embryonic development is entirely under control of maternal gene products. Therefore, all stage 2 transcriptomes are supplied entirely by XX genomes and the zygotic genome is transcribed by either XX or XY genomes. Given the possibility of different evolutionary pressures, we asked whether there is a difference of enrichment of DE genes on the autosomes or X chromosome across maternal and zygotic stages. For our stage 5 transcriptomes, these were collected from XY embryos, so they are directly comparable. As onset of Drosophila dosage compensation occurs sometime after stage 5, collecting a single sex is necessary at this stage [20, 21, 22]. We normalized the number of DE genes per chromosome by the number of genes expressed on each chromosome.

We found that DE genes at stage 2 between populations were enriched on the X chromosome compared to the autosomes (Fig. 3B). However, enrichment of DE genes on the X chromosome is absent at stage 5 in our samples. Maternal transcripts are not completely degraded by stage 5, so we also asked if the trend seen for all of stage 5 transcripts were the same for transcripts that are zygotic-only. As expected, fixed expression differences between zygotic-only genes were not enriched on the X chromosome (Fisher’s exact test, *p* < 0.05) having the same result as all genes at stage 5.

### The most differentially expressed genes are genes with known selection signatures

**Fig. 5:**
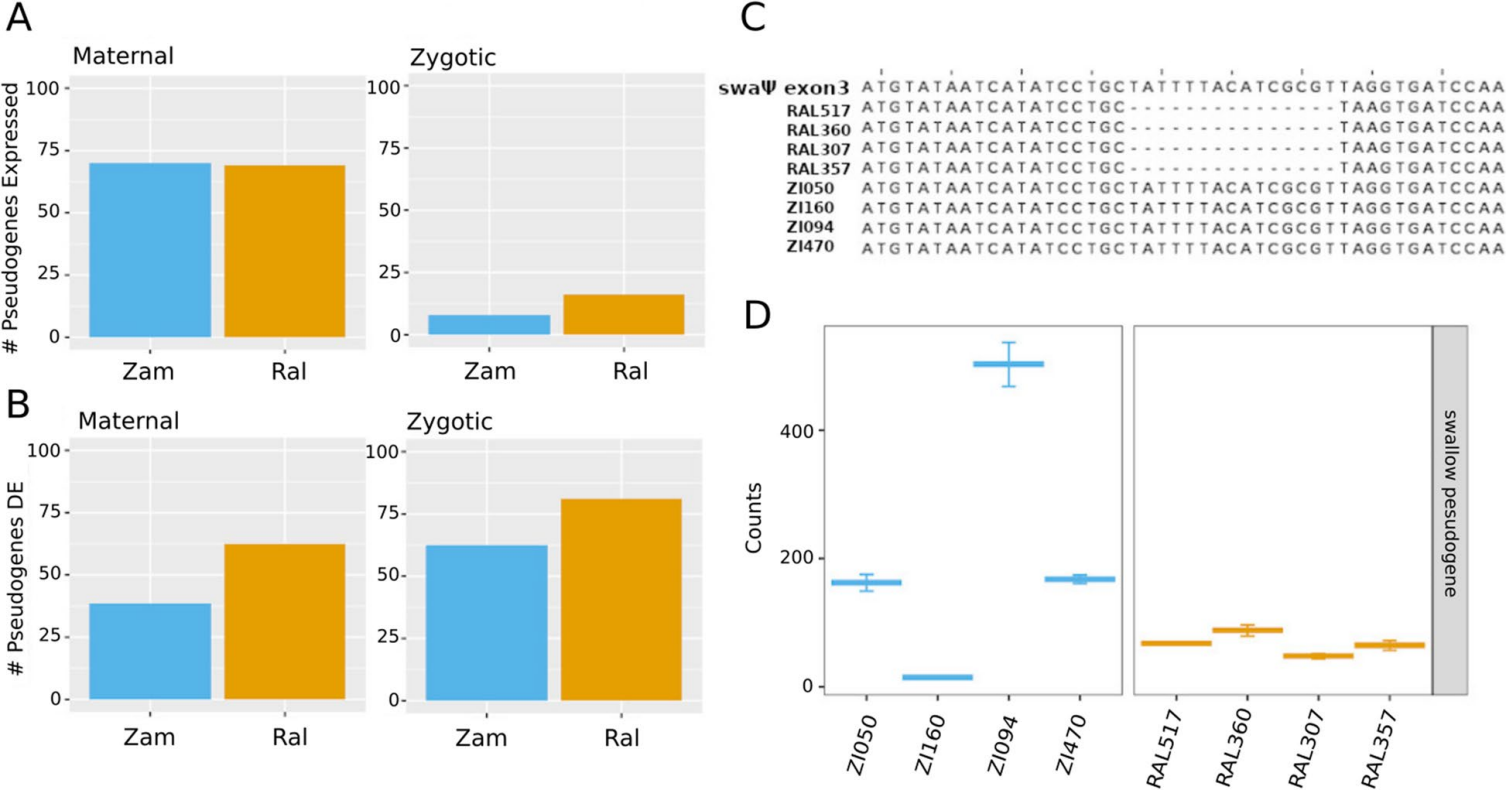
Examples of differentially expressed genes with previous evidence for functional significance. **A** Transcript levels for three example genes, shown at both developmental stages labeled across the top, for the Raleigh lines (orange) and the Zambia lines (blue). **B** Results of experiments testing survival of embryonic heat shock across lines, showing relative survival at three temperatures. While on average the Raleigh lines have higher survival after heat shock at 24 °C and 38 °C, they also have higher survival at standard rearing temperatures, results do not correspond well with heat shock transcript levels

A number of the most differentially deposited transcripts between populations are genes that have been shown previously to have signatures of selection at the level of the genome under different conditions. For example, a previous study found that genes within the chemosensory system have undergone local adaptation following *D. melanogaster*’s global expansion out of Africa [23]⁠⁠. This study was based on the genomes of five different geographically distinct populations of *D. melanogaster* including both North American and African populations. Notable within the top ten most DE maternally deposited genes between populations is *Gstd9*, a glutothione-S-transferase, which belongs to a gene family that was found to have signals of selective sweeps upon global expansion ⁠⁠[23]⁠. In total, seven glutothione-S-transferases were found to be differentially deposited between the Raleigh and Zambia populations. In the same study [23]⁠⁠ the zinc finger protein family was shown to have strong population differentiation. *Zcchc7*, a zinc-finger protein, is also among the top ten most differentially deposited transcripts. These two genes both have undergone dramatic qualitative changes in maternal deposition (Fig. 3A, Fig. 5A and Supplemental Table 1).

The second most significantly differentially deposited transcript is the actin binding protein *Unc-115a*. The paralog of this gene, *Unc-115b*, was also found to be differentially expressed between populations. Both of these genes have higher expression levels in the Raleigh population. *Unc-115b* was found in a previous study to be the most highly upregulated gene in a *D. melanogaster* strain resistant to the insecticide DDT 91-R compared to a DDT compromised strain, 91-C ⁠[24]⁠. *Unc-11b* was one of two genes found in this study to be highly upregulated across all stages of development that were assayed [24]⁠. This gene was found to be in one of six selective sweeps that coincided with constitutive expression differences between DDT resistant and compromised lines.

### Variation in heat shock proteins

Modifying maternal RNAs and proteins in the embryo can have effects on development, phenotypes, and ultimately fitness [25, 26]⁠⁠⁠. One gene family that is critical to survival is heat shock proteins [27, 28]⁠. In total, 17 and 19 heat shock proteins were found to be differentially deposited within the Raleigh population and Zambia population, respectively. This is in contrast to after zygotic genome activation, where 6 and 8 zygotic-only heat shock proteins were found to be differentially expressed within the two populations. Previous work by Lockwood et al. has shown evidence that higher levels of maternal deposition of a heat shock protein increases embryo thermal tolerance in *D. melanogaster* [29]⁠⁠. *Hsp23* was found to be differentially deposited in the lines that we examined (Fig. 5A, bottom panel). Specifically, the levels of *Hsp23* mRNA in ZI094 is between 4-14X higher than the other three Zambia lines and 11-600X higher levels than the Raleigh lines, all which have variable expression. This overall trend persists at stage 5, with mean levels of *Hsp23* increasing in ZI094 and maintaining higher expression levels compared to all other lines. Based on this observation, we performed heat shock experiments on all of the lines to assay differences in embryo survival after heat stress (see Methods). We did not find a significant relationship between line and survival (ANOVA, *p* < 0.05) at 24 and 36 degrees, however there was a small significance (*p* = 0.014) at 38 degrees. However, we found that heat shock tolerance does not correspond in a predictive way with levels of heat shock transcripts (Fig. 5B).

### A number of the most differentially expressed genes are annotated as pseudogenes

**Fig. 6:**
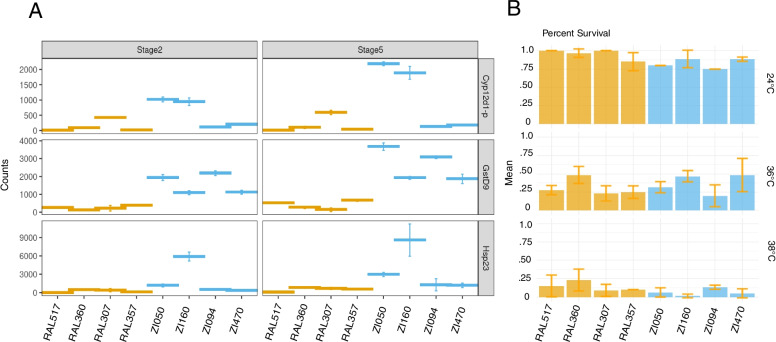
Variation in pseudogene transcript levels. **A** At each stage (maternal, zygotic), the number of annotated pseudogenes expressed are similar between populations. The smaller number of pseudogenes expressed at the zygotic stage reflect that this analysis was restricted to zygotic-only genes, which are zygotic genes with no maternal expression. **B** Of the pseudogenes expressed at each stage, a larger proportion are differentially expressed in the Raleigh lines. **C** One example, the *swallow* pseudogene*,* has a 15 bp deletion shared by all of the Raleigh lines at the position shown in the alignment. **D** The *swallow* pseudogene is more highly expressed in a number of the Zambia lines, with considerable variation between lines

The most differentially maternally deposited gene between the Zambia and Raleigh populations in our analysis is the gene *CR40354* which is annotated in the *D. melanogaster* genome as a pseudogene with unknown function. This prompted us to investigate other genes annotated as pseudogenes in our dataset because previous annotations that identified these genes as pseudogenes were more likely to have been done in non-African populations. We asked how many pseudogenes were maternally deposited and zygotically expressed within and between populations. A total of 69 and 70 genes labeled as pseudogenes were found to be maternally deposited within the Raleigh and Zambia populations, respectively (Fig. 6A). A total of 16 and 8 genes labeled as pseudogenes were found to be expressed from the zygotic genome but not the maternal genome (zygotic-only, see Methods) in the Raleigh and Zambia populations. Between the populations, 18 pseudogenes were found to be differentially maternally deposited and 16 of the zygotic-only pseudogenes were found to be differentially expressed at stage 5 (Fig. 6B). One pseudogene which caught our attention was the *swallow Ψ* (*swaΨ*) pseudogene which is differentially expressed within the Zambia population in our analysis. *swaΨ* is a result of a recent genome duplication of *swallow*, and is only found in *D. melanogaster* [30]⁠⁠⁠. *swallow* is a critical gene to early development, and is required for proper Bicoid positioning in the embryo [31]⁠. Previous studies [32]⁠⁠ have suggested that *swaΨ* not transcribed in *D. melanogaster*. We found it to be very lowly expressed in the Raleigh lines, but variably expressed within the Zambia lines with one line, ZI160, showing relatively high expression levels (Fig. 6D). To investigate further, we sequenced the swaΨ locus in each of the lines. We discovered a 15 bp population-specific deletion present (Fig. 6C). All Raleigh lines have a 15 bp deletion in the annotated exon 3 of *swaΨ*, which is not present in all four Zambian lines. This sequence is part of the fully functional exon 3 of the *swallow* gene.

## Discussion

Gene expression is a multi-step process that is fundamental to all cellular activities. In Drosophila, RNA transcription is highly regulated during oogensis and embryogenesis, and this precision in transcription regulation is critical for proper development of the embryo. While previous studies have shown that the maternal and early zygotic transcriptomes are highly conserved across species ⁠[6, 33, 34]⁠, here we show that that there is variation present in gene expression on the shorter evolutionary timescale represented within a species, *D. melanogaster*. We chose lines from Siavonga, Zambia and Raleigh, North Carolina, USA to encompass a broad span of genetic diversity within and among populations (Fig. 1).

Our results show that the transcriptomic dynamics at these developmental stages reflect what is known about the population genetic history of *D. melanogaster* from genomic studies. Previous studies found more genetic variation within African populations than non-African populations [7, 8, 15, 35]⁠ and⁠ we found the same pattern with transcript levels from maternal and early zygotic transcriptomes (Fig. 2A). There are differential transcript abundances within both the Zambia and Raleigh populations, and some of the same transcripts are variable within each population, but there is more population-specific variation within the Zambia lines. We also find that with pairwise comparisons between lines, the Raleigh lines have far fewer genes identified as differentially expressed, but comparisons within Zambia have as many (stage 2) or only slightly fewer (stage 5) differentially expressed genes as when comparing lines from the two populations. The increased number of differentially expressed genes in the Zambia lines is consistent with high levels of genomic variation found in the ancestral range of this species [35]⁠⁠. And the reduced number of differentially expressed genes in Raleigh likely reflects the lower genetic polymorphism levels following the out-of-Africa bottleneck [7, 36]⁠⁠. Interestingly, while consistent with the genomic variation within these lines, our results stand in contrast to microarray studies in adults which found less transcript variation within African and non-African populations than between, which has been taken as a sign of directional selection ⁠⁠[13, 37]⁠.

Also consistent with previous genomic studies are the numbers of genes highlighted by our DE analysis that have also been identified in studies performing artificial selection or population genomic studies on the global expansion of the species [23, 24]⁠⁠. Many are used as examples throughout the manuscript and have been associated with xenobiotic metabolism (*GstD9*, and *Cyp12d1-p*), possible environmental adaptation to global expansion (*Zcchc7*), and DDT resistance (*Unc-115a* and *Unc-115b*). Thus, many of our most significantly DE genes are also likely under selection, and their functions are consistent with adaptation to a new environment. Studies to determine the adaptive function of these genes are often carried out in adults [38, 39]⁠⁠⁠ but our data suggests that these differences in transcript level are also present in the embryo, and thus may potentially be of adaptive value at this stage.

We find a stage 2-specific enrichment of differentially expressed genes between the Zambia and Raleigh populations on the X chromosome (Fig. 3B). Previous studies have shown a reduction in heterozygosity on the X chromosome relative to the autosomes in temperate European populations compared to populations from sub-Saharan Africa. This reduction in heterozygosity has been attributed to demographic events following the out-of-Africa expansion of *D. melanogaster* ⁠[12]⁠. Therefore, it is possible that the decreased heterozygosity on the X chromosome has led to decreased differences in transcript levels of genes on the X within the Raleigh populations. This decrease in expression variation within Raleigh may contribute to the strong signal of between population differences in expression we find specifically on this chromosome. However, this pattern of enrichment is only seen at stage 2, where all transcripts are from the maternal, XX, genome, and therefore may be under unique selective pressures.

Among the genes we found to be differentially expressed were heat shock proteins, including *Hsp23*. *Hsp23* has previous evidence of increasing embryo heat tolerance when maternally loaded [29]⁠. Here, we adapted the same heat shock and embryo lethality protocol to determine differences in thermotolerance between lines at stage 2 (Fig. 5B). We did not find such a linear relationship between thermotolerance and maternal *Hsp23* levels at varying temperatures of heat shock. Our results differ from those found in a previous study [29], which can likely be explained due to differences in study design. In this previous study [29], researchers overexpressed *Hsp23* in lines of the same genetic background. In this study, we use lines from different populations that may therefore have expression variation of genes that may affect thermotolerance. In fact, we have found differential expression in over 30 heat shock proteins at stage 2 within the two populations as well as glutathione s-transferases which have separately shown to have roles in thermotolerance [40]⁠⁠. It is possible that more complex interactions among the genes in these networks underlie the patterns of thermotolerance we find in these lines across temperatures.

Genes annotated as pseudogenes were called significantly differentially expressed in our analysis both within and between populations. Most striking is the fixed expression difference of the *swallow pseudogene* (*swaΨ)* between populations at stage 2 (Fig. 6D). *swaΨ* is the result of a relatively recent duplication of the *swallow* gene which is maternally expressed and required for proper anterior–posterior axis patterning. Genome-wide analysis of pseudogenes in *D. melanogaster* has shown that *D. melanogaster* have relatively low proportion of pseudogenes (110 were identified in one study [41]⁠⁠), with respect to their proteome, compared to other eukaryotic genomes such as human, nematode and budding yeast [41]⁠⁠. It has been speculated that the low number pseudogenes suggests a high rate of DNA loss in *Drosophila* ⁠[32]⁠. Here, we find that *swaΨ* has most likely acquired a 15 bp deletion after the migration of *D. melanogaster* out of Africa (Fig. 6C). We also find that *swaΨ* is expressed in a number of the Zambia lines but very low to no expression was detected in any of the Raleigh lines. This data suggest that in addition to deletions *swaΨ* has also lost maternal expression over time.

## Conclusions

Previous studies have found an especially high degree of conservation of the maternal transcriptome across species [6, 33, 34]⁠; this study provides evidence this is also true within *D. melanogaster*. Whether examining the number or proportion of differentially expressed genes within populations, between populations, between pairs of lines, or between species, there are fewer differences in transcript levels found at stage 2, when all transcripts are maternal, than at stage 5, after zygotic genome activation. The analysis of proportions of genes DE within and between species is especially suggestive relative to these stage-specific dynamics. At stage 5, the proportion of genes DE between species is far higher than the within-*D. melanogaster* comparisons, and there is a higher proportion of DE genes overall in every comparison. In contrast, at stage 2, there are fewer genes DE in each comparison, and the between species comparisons (while still higher on average than the within-*D. melanogaster* comparisons) are only slightly higher. This suggests that relative to one another, more of the maternal transcriptome may be under stabilizing selection than the more rapidly evolving zygotic stage transcriptome [42]⁠⁠⁠.

In conclusion, we find that the maternal and zygotic transcriptomes, while generally conserved, do show some interesting differences in transcript abundance even in the relatively short period of evolutionary time represented by the diversity within a species. This species, *D. melanogaster,* has more variation in transcript abundance at these critical developmental stages within populations than between them. And consistent with what has been determined between Drosophila species [6]⁠⁠, we show that the maternal transcriptome is more highly conserved than the zygotic transcriptome, and more of the maternal genome may be under purifying selection. Together, the presented data highlight how a constrained developmental trait evolves over short periods of evolutionary time.

## Methods

### Single Embryo collection and sequencing library generation

Fly lines from Siavonga, Zambia (courtesy of the Langley Lab, University of California, Davis) were isofemale lines inbred for at least 5 generations [15]. The North American lines are from the Drosophila Genome Reference Panel [16]. They were collected as isofemale lines, from Raliegh, NC, and inbred for 20 generations [16]. All fly lines were population controlled on cornmeal fly food at 25**°**. Four lines from Zambia (ZI050, ZI094, ZI160, and ZI470) and four lines from Raleigh (RAL307, RAL357, RAL360, and RAL517) were selected for embryo collection. Single embryo RNA extraction was adapted from Lott et al., 2011 [5]. Embryos were dechorionated using 50% bleach for 2 min. Embryos in halocarbon oil were imaged using a Zeiss Axioimager. Embryos were staged based on morphology. Stage 2 embryos were chosen based on observation of retraction of the vitelline membrane from both the anterior and posterior poles, before the pole cells were visible. Late stage 2 embryos were chosen based on cellularization having just been completed, prior to the beginning of gastrulation. Methods for single embryo RNA extractions were performed as in Lott et al., 2011 and Atallah et al., 2018 [5, 6]. After imaging, embryos were moved from the microscope slide, rolled into a drop of Trizol (Ambion), and the viteline membrane ruptured with a needle. The single embryos now in drops of Trizol were then moved to a tube with additional Trizol, then frozen at -80 ° C until extraction. Nucleic acids (both RNA and DNA) were extracted according to manufacturers’ protocol, except using a more Trizol reagent than specified (1 mL) given the expected concentration of DNA and RNA as in Lott et al. 2011 [5].

Since embryos were collected from a large number of mothers, it is unlikely that multiple samples came from the same mother. Stage 2 and late stage 5 embryos were identified based on morphology. Stage 2 embryos were selected based on the vitelline membrane retracting from both the anterior and posterior poles, prior to when pole cells become visible. Late stage 5 embryos were chosen based on having completed cellularization, but not yet having started gastrulation. Embryos were then removed from the slide with a brush, cleaned of excess oil, placed into a drop of Trizol reagent (Ambion), and ruptured with a needle, then moved to a tube with more Trizol to be frozen at -80 ° C until extraction. RNA and DNA were extracted from single embryos as in the manufacturer’s protocol, with the exception of extracting in an excess of reagent (1 mL was used) compared to expected mRNA and DNA concentration. Extracted DNA for stage 5 embryos was used for genotyping for sex as in Lott et al., 2011, XY embryos were selected for transcriptomic analysis, due to the incomplete nature of X chromosomal dosage compensation in XX embryos at this stage [5]⁠⁠.

RNA-Seq libraries were prepared for single embryos using poly-A enrichment for each of the 8 lines (4 Zambia lines and 4 Raleigh lines), for both stage 2 and stage 5, with 3 replicates each, for a total of 48 libraries. These samples were sequenced 100 bp, paired-end, on an Illumina HiSeq4000. The sequencing was carried out by the DNA Technologies and Expression Analysis Core at the UC Davis Genome Center, supported by NIH Shared Instrumentation Grant 1S10OD010786-01.

### Data processing

Reads were trimmed and adapters removed using Cutadapt version 1.7 [43], and gently (PHRED Q < 20) trimmed for quality [44]⁠. Mapping was done with the *D. melanogaster* Flybase genome release 6.18 and associated annotation file using HISAT2 version 2.1.0 ⁠[45]⁠ using default parameters. Gene level counts were generated using featureCounts of the subRead [46]⁠ package in R [47]⁠ (R version 3.4.1). Counts were normalized to sequencing depth and RNA composition using DEseq2’s median of ratios. Count data can be found in Supplemental File 2. Overall experimental worflow is diagrammed in Supplemental File 1.

### Data availability

All raw and processed data are available at NCBI/GEO under accession number GSE195496. Processed data (transcript level counts) are also available in Supplemental File 2.

### Hierarchical Clustering and PCA analysis

We performed hierarchical clustering analysis in R using the hclust function. A dissimilarity matrix (*dist()*) of one minus the Spearman correlation (*cor()*) was used for hierarchical clustering. Principal component analysis (PCA) was also performed in R using the *prcomp()* function.

### Determining on or off State

To determine whether a gene was likely to be transcribed based on the count data, we ran Zigzag [48]⁠ on our data. A full description of how this program was utilized, see Supplemental File 3.

### Differential expression analysis

Differential expression analysis was done using the DEseq2 (version 3.1) ⁠[49]⁠ package in R. Using DEseq2, we implemented the LRT (likelihood ratio test). For within- population analysis the replicates for each line were given the same label for the design matrix. For determining the differences between populations, we labeled lines as either Raleigh or Zambia in the design matrix and implemented the LRT test. DEseq2 results for within and between populations are included in Supplemental File 4. When comparing the number of DE genes within and between populations, the number of DE genes is divided by the number of genes expressed in order to compare % DE genes between stages. We counted a gene as expressed in the total number of genes expressed for normalization if the gene was expressed in at least one line, as described above.

For pairwise differences between lines, DEseq2 was run on every possible combination of pairs. Since there are more between population pairs than within population pairs, we ran bootstrapping in R in order to compare the number of DE genes between lines of the same population and between lines of different populations. To test if the distributions of bootstrapped averages were significantly different from one another, we implemented a Wilcoxon rank sum test in R. When plotting the magnitude of differences between differentially expressed genes we used the foldchange2logratio function in R to compute log-ratios from fold-change values.

For differential expression analysis between species we used RNA-seq data previously generated in the lab [6]⁠⁠ ⁠from *D. simulans, D. sechellia, D. erecta,* and *D. yakuba.* Reads were aligned using HISAT2 [45] followed by FeatureCounts [46] to generate expression levels in counts. Counts were then normalized using the *norm()* function in DESeq2. Only genes which had orthologs in all seven species were considered. We used the ortholog table (dmel_orthologs_in_drosophila_species_fb_2019_03.tsv.gz) downloadable from Flybase to determine which genes had orthologs in all seven species. An expression cutoff of 3 counts was used to determine which genes were considered expressed in each line.

### Test of enrichment on autosomes or sex chromosomes

To determine whether there was enrichment of DE genes on either the autosomes or sex chromosomes the chromosomal location of each DE gene was determined. Number of DE genes per chromosome was normalized to the number of genes expressed on the chromosome. We implemented a Fisher’s exact test in R to determine if there is a significant difference in how many DE genes are on autosomes compared to the X chromosome. This was performed by doing individual tests between the number of DE genes on each autosome and the X.

### Heat shock of embryos

We adapted the heat shock and embryo survival protocols from [29]⁠. Flies aged 3–5 days were allowed to lay eggs on a clearance plate for one hour. Plates were then swapped with clear agar collection plates with additional yeast and flies allowed to lay for an additional hour in order to collect 0–1 h aged embryos. Plates were then wrapped in parafilm and fully submerged in a heat bath at the given temperature (either 24**°**, 36**°**, or 38**°**) for 40 min. Embryos were then grouped in a line of 20 embryos using a brush. Proportion of embryos hatched was assayed 48 h after heat shock to determine embryo survival. Three temperatures were assayed.

## Supplementary Information

**Additional file 1. **Flow chart of general experimental work flow of RNA extraction and DE analysis.

**Additional file 2. **Gene level count data for all embryos used in the analysis generated using featureCounts.

**Additional file 3. **Description of the implementation of ZigZag for determining which genes are expressed in the embryos.

**Additional file 4. **Results showing differentially expressed genes within Zambia and Raleigh populations and between the two populations at both stage 2 and stage 5. Differential expression analysis was done using the DEseq2 (version 3.1) ⁠[49]⁠ package in R. Using DEseq2, we implemented the LRT (likelihood ratio test). For within- population analysis the replicates for each line were given the same label for the design matrix. For determining the differences between populations, we labeled lines as either Raleigh or Zambia in the design matrix and implemented the LRT test.

**Additional file 5: Table S1.** Top ten differentially expressed genes (DESeq2) between Zambia and Raleigh populations at stage 2 and stage 5.

## Data Availability

All sequencing data and processed data files from this study are available at NCBI/GEO at accession number: GSE195496.

## Abbreviations

MZT: Maternal to zygotic transition
DE: Differential Expression
DGRP: Drosophila Genetics Reference Panel
