# Supplementary material for: Natural variation in the maternal and zygotic mRNA complements of the early embryo in Drosophila melanogaster
Source: BMC Genomics. 2022 Sep 8;23:641. doi: 10.1186/s12864-022-08839-4 (PMC9461177; doi:10.1186/s12864-022-08839-4)
Supplement: Supplementary file 1 — Additional file 1. Flow chart of general experimental work flow of RNA extraction and DE analysis. [file 12864_2022_8839_MOESM1_ESM.pdf]

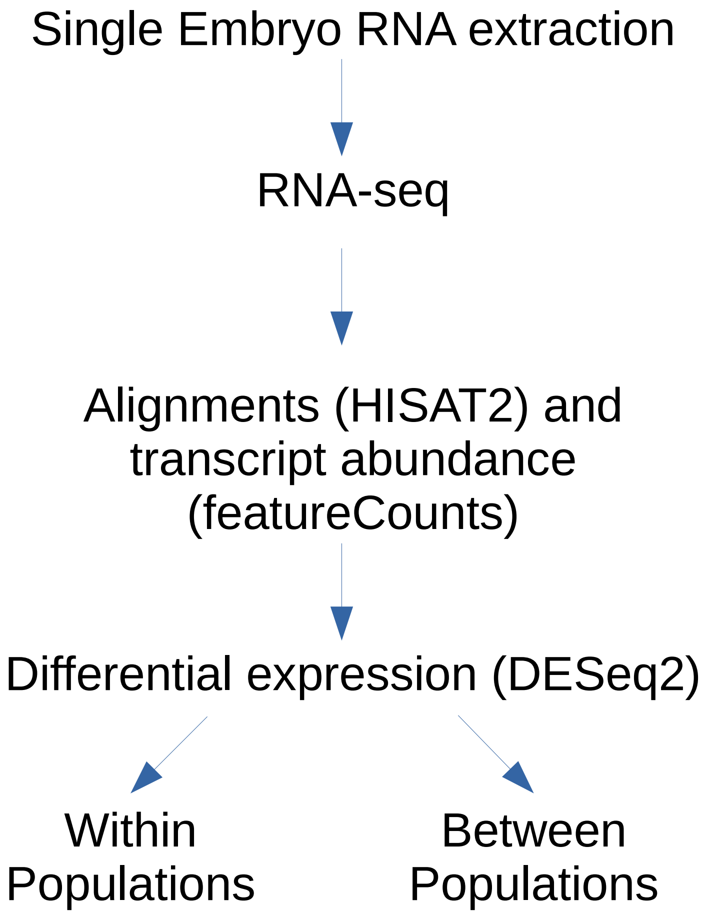
Single Embryo RNA extraction
RNA-seq
Alignments (HISAT2) and transcript abundance (featureCounts)
Differential expression (DESeq2)
Within Populations
Between Populations
