# Supplementary material for: Natural variation in the maternal and zygotic mRNA complements of the early embryo in Drosophila melanogaster
Source: BMC Genomics. 2022 Sep 8;23:641. doi: 10.1186/s12864-022-08839-4 (PMC9461177; doi:10.1186/s12864-022-08839-4)
Supplement: Supplementary file 3 — Additional file 3. Description of the implementation of ZigZag for determining which genes are expressed in the embryos. [file 12864_2022_8839_MOESM3_ESM.pdf]

Zigzag analysis

Data processing

The expression states of all mRNA encoding genes were analyzed using the R package zigzag (Thompson et al. 2020). Zigzag requires at least two replicate libraries of relative expression levels for each gene as well as the mean or median transcript length of each gene. The Drosophila melanogaster annotation for the reference genome assemly, r6.18 was used to obtain mean transcript length estimates for protein coding genes. This was done using a custom bash script available here: https://github.com/ammonthompson/zigzag/tree/master/scripts.

In some of the lines, a small number of genes exhibited extreme variation among replicates. These outlier genes exhibited poor mixing in the zigzag MCMC. As a result, despite having very high expression (all replicates > 20 TPM), zigzag assigned inconsistent estimates among independent runs with surprisingly low probability of active expression for these genes. Rather than removing these high-expressed outliers from further analysis, we set their prior probability of being active to 1. This significantly improved MCMC mixing.

We detected outliers by fitting a linear model to the log-standard deviation to log mean of gene expression across replicates for genes which had greater than 20 TPM in all replicates. For each dataset, we defined an outlier by finding the 10% FDR threshold assuming Normally distributed residuals. We confirmed that this is approximately true by analyzing quantile-quantile plots of the residuals. 0 - 17 genes in each stage 2 library were flagged as outliers and assumed active using this approach.

Mixture model and MCMC settings

All zigzag analyses were performed on a high performance computing cluster maintained by the University of California, Davis Bioinformatics Core. Table S.AT.1 shows the hyper-prior settings for the selected mixture model for gene activity state inference. Prior distributions for mixture component means were set to have non-overlapping regions to impose identifiability constraints on the posterior distribution. For both stages, the data clearly exhibit two large overlapping peaks where an upper bound for the inactive component mean prior and the lower bound of the active component mean prior was set. An additional small population of very highly expressed genes was consistently observed in the right tail of the data distribution (see figure S.AT.1 below).

Figure S.AT.1: Transcriptome distributions. Example transcriptome expression distribution for a stage 2 and a stage 5 sample. Vertical lines

show the lower and upper boundaries for the mixture component means of the active and inactive subcomponent prior distributions respectively.

png 2

The number of components for a given mixture model was selected after performing posterior predictive checks for a model with one inactive component and one, two, or three active components (see “Mixture Model checking and selection” section below).

Hyperparameter settings

	stage 2	stage 5
s0_mu	-1	-1
s0_sigma	2	2
s1_shape	1	1
s1_rate	2	2
tau_rate	1	1
tau_shape	1	1
alpha_r_shape	1	1
alpha_r_rate	0.1	0.1
weight_active_shape_1	2	2
weight_active_shape_2	2	2
weight_within_active_alpha	0.667	0.667
spike_prior_shape_1	1	1
spike_prior_shape_2	1	1
active_means_dif_prior_shape	1	1
active_meaans_dif_prior_rate	0.3333	0.3333
active_variances_prior_min	0.01	0.01
active_variances_prior_max	10	10
inactive_means_prior_shape	1	1
inactive_means_prior_rate	0.3333	0.3333
inactive_variances_prior_min	0.01	0.01

inactive_variances_prior_max	15	15
threshold_i	-1	2
threshold_a	-1, 3, 5	2, 5

MCMC

Zigzag simulates draws from the posterior distribution using Markov Chain Monte Carlo (MCMC). Four independent MCMC chains were run for each line. Burn-in was run for 20000 zigzag generations. If convergence was achieved, then the mcmc was run for an additional 1 million generations, while sampling every 100 generations. One of the four runs also sampled from the posterior predictive distribution periodically during the MCMC.

MCMC diagnostics

Trace plots indicate all independent runs converged on the same stationary distribution and mixed well. To examine prior sensitivity, the marginal posterior distributions for each model parameter was compared to their prior distribution. The marginal posteriors showed very low variance relative to the local prior's variance and were distant from prior boundaries indicating very little influence of the prior distribution on the posterior.

The autocorrelation-corrected posterior sample size which is called the effective sample size (ESS) of each simulated sample from the posterior was measured using the function *effectiveSize* from the R package *coda.* All hyperparameter's had ESS >> 200. ESS was also measured for gene-level parameters; the true mean and variance of each gene. If a gene's ESS was less than 100 in one of the four chains for either of these parameters, the gene was flagged. Each chain only had a few genes with ESS < 100. Despite this somewhat low sample size, these genes had very similar posterior probabilities of active expression. In all, less than 12 genes were flagged per line analyzed.

Convergence to the same stationary distribution for the four chains was assessed by measuring the potential scale reduction factor, or PSRF (Brooks and Gelman 1997). We used the function *gelman.diag*, also from *coda*, setting the transform argument to TRUE and the multivariate argument to FALSE. We determined that the chains converged if PSRF was close to 1 (< 1.2).

Mixture Model checking and selection

Model adequacy was assessed using posterior predictive simulation. During the MCMC, zigzag periodically simulates a dataset given the posterior sample, which is compared to the real dataset by measuring three discrepancy statistics and computing the difference in these statistics

between the simulated and real data (Thompson *et al*. 2020). If the distribution of differences measured from simulated data and the real data overlaps with zero then that is an indication that the fit model can generate data that is similar to the data used for inference. We used this approach, as well as visually comparing simulated and real data distributions (see below), to determine how many mixture components are necessary to achieve a good fit to the data.

In brief, posterior predictive simulation is done by drawing mixture model parameters from the joint posterior distribution, using those parameters to simulate new data under the model, measuring discrepancy statistics with the simulated data and comparing those statistics to the same statistics measured from the real data. If simulated data resembles the data then the discrepancy statistics will be similar and the difference between them will be close to zero.

Posterior predictive simulation indicated that a three component mixture model (one inactive and two active mixture components) yields a good fit for both stages (Figures S.AT.2 and S.AT.3). The posterior predictive distributions for stage 2 was greatly improved when an additional mixture component was included (four total).

Figure S.AT.2: Stage 2 posterior predictive discrepancy statistics. Box and whisker plots show the data and interquartile ranges for three component (blue) and four component (red) models fit to the stage 2 data for the three discrepancy statistics from Thompson et al. (2020) measured from 2550 posterior predictive simulations.

```
## png
##   2
```

We attempted to fit a similar number of mixture components to the stage 5 distribution where the inactive and active components show much more overlap. Likely because of the greater overlap, we were unable to fit this more complicated model using zigzag and therefore use the two active component model for stage 5.

Figure S.AT.3: Stage 5 posterior predictive discrepancy statistics. Box and whisker plots show the data and interquartile ranges for three component stage 5 data for the three discrepancy statistics from Thompson et al. (2020) measured from 2550 posterior predictive simulations.

```
## png
##   2
```

We also overlaid predictive distribution of simulated transcriptome distributions with the posterior distribution observed in the data to further assess fit and locate regions of the distribution where fit could potentially improve by further modification to the model. We found that the

distributions of simulated data overlapped over much of the real data distribution for all of the lines analyzed with zigzag. Figure S.AT.4 shows the predictive densities of true expression levels and posterior densities of true expression levels for a model with one, two and three active components. The figure shows how increasing the number of mixture components gradually increases the overlap between the predictive distribution and the posterior distribution. The distance between the two is measured by the upper level Wasserstein metric “W_U” above. See the supplemental appendix in Thompson et al. (2020).

Figure S.AT.4 Example posterior predictive distribution of latent mixture model fit by zigzag with one, two, three or four components. Simulated distributions of true gene expression levels, (P(Y’|X); green) overlaid with the posterior distribution of true expression levels,(P(Y|X); orange). X is the data, Y is the true, unobserved expression levels of genes, and Y’ is the predicted true expression level of genes for simulated data. Arrows point to approximate location of mixture component means.

Final probability estimation

After confirming all chains converged onto the same joint posterior distribution, all four chains were concatenated to get a combined posterior estimate of each gene’s activity state. If individual gene-level parameters (means and variances) had ESS < 100 in any of the four chains then that gene was flagged. Ten genes were flagged in at least one line. All but three of those genes had identical posterior probabilities of active expression in all lines. Figure S.AT.5 shows examples of the expression probabilities estimated by zigzag compared to the expression distributions (log TPM) for a single library from a stage 2 and a stage 5 sample.

Figure S.AT.5: Probabilities of 500 random genes with respect to their log TPM in estimated from a single library of a stage 2 data set and a stage 5 data set.

```
## png
##   2
```

Zigzag MCMC and posterior predictive simulation files are located in supplemental data file zigzag_DATA.AT.1.tar.gz
